# Toxicity and bioaccumulation of Cadmium, Copper and Zinc in a direct comparison at equitoxic concentrations in common carp (*Cyprinus carpio*) juveniles

**DOI:** 10.1371/journal.pone.0220485

**Published:** 2020-04-09

**Authors:** Vyshal Delahaut, Božidar Rašković, Marta Satorres Salvado, Lieven Bervoets, Ronny Blust, Gudrun De Boeck

**Affiliations:** 1 Department of Biology, University of Antwerp—Faculty of Sciences, Antwerp, Belgium; 2 University of Belgrade—Faculty of Agriculture, Institute of Animal Science, Zemun, Belgrade, Serbia; Chinese Academy of Sciences, CHINA

## Abstract

The individual toxicity and bioaccumulation of cadmium, copper and zinc for common carp juveniles was evaluated in a direct comparison in two experimental setups. First, fish were exposed for 10 days to different metal concentrations in order to link metal bioaccumulation to LC_50_ values (concentration lethal to 50% of the animals) and incipient lethal levels (ILL, concentration where 50% survives indefinitely). Accumulated metals showed a positive dose dependent uptake for cadmium and copper, but not for zinc. Toxicity was in the order cadmium>copper>zinc with 96h LC_50_ values for cadmium at 0.20±0.16 μM, for copper at 0.77±0.03 μM, and for zinc at 29.89±9.03 μM respectively. For copper, the 96h exposure was sufficient to calculate the incipient lethal level and therefore 96h LC_50_ and ILL levels were the same, while for cadmium and zinc 5 to 6 days were needed to reach ILL resulting in slightly lower values at 0.16 μM and 28.33 μM respectively. Subsequently, a subacute exposure experiment was conducted, where carp juveniles were exposed to 2 equitoxic concentrations (10% and 50% of LC_50_ 96 h) of the three metals for 1, 3 and 7 days. Again a significant dose-dependent increase in gill cadmium and copper, but not in zinc, was observed during the 7-day exposure. Copper clearly affected sodium levels in gill tissue, while zinc and cadmium did not significantly alter any of the gill electrolytes. The overall histopathological effects (e.g. hyperemia and hypertrophy) of the metal exposures were mild for most of the alterations. Our study showed that copper an cadmium (but not zinc) showed dose dependent metal accumulation, however this bioaccumulation was only correlated with mortality for cadmium. Metal specific alterations were reduced gill sodium levels in copper exposed fish and oedema of the primary epithelium which typically occurred in both levels of zinc exposure.

## Introduction

Anthropogenic input of metals remains one of the major threats to aquatic animals and entire aquatic ecosystems. These contaminants may originate from a wide range of sources, i.e. industrial activities, traffic, households or agriculture [1, 2]. Amongst others, cadmium (Cd), copper (Cu) and zinc (Zn) are three metals which are often encountered in surface waters at levels exceeding the environmental quality standards set by the European Union [3]. The EU Directive is classifying inland surface waters into 5 classes, depending on various pollutants and other water parameters [3], and those standards are incorporated in water policy laws of EU member states. Maximum allowable concentrations (MACs) for cadmium are given in the Directive (from Class I <0.45 μg/L to Class V >1.2 μg/L). MACs for copper and zinc are not given in the Directive, however, the Organisation for Economic Co-operation and Development [4] published a guideline, where MACs for each class of surface waters can be found. Those MACs vary for copper from Class I <20 μg/L to Class V >100 μg/L; and for zinc from Class I <45 μg/L to Class V >120 μg/L, reflecting the range of existing concentrations in surface waters. In Belgium, where this study was conducted, the latest report mentioning total concentrations of cadmium, copper and zinc measured by the Flemish environmental agency (VMM) in 2014, ranged respectively from 4.45e^-4^–0.03 μM (0.05–3.37 μg/L), 0.02–0.54 μM (1.27–34.32 μg/L) and 0.12–5.05 μM (7.84–330.17μg/L) [5], ranking some of these rivers in Class V. However, exceedances of these levels in both surface an drinking waters have been reported worldwide [6, 7, 8].

Fish living in metal polluted environments might either be exposed to metals through the food chain, or via direct uptake from contaminated water. In the latter case, the gills are the first organs to suffer from this kind of pollution and will show the first clinical signs induced by waterborne metal exposure [9]. Copper and zinc are essential nutrients for fish, and therefore dietary or waterborne intake of these elements is necessary to sustain basic metabolic processes, in contrast to xenobiotic metals such as cadmium [1]. However, elevated concentrations of copper or zinc will also lead to adverse effects on a wide range of crucial pathways.

Copper uptake is facilitated via two distinctive mechanisms: a transmembrane protein (copper transporter 1), insensitive to external copper concentrations, and the apical Na^+^-uptake pathways located at branchial epithelial cells, sensitive to external concentration of copper [10, 11]. In the latter case, intracellular sodium levels can decrease as a direct consequence of competition at the uptake site [12]. In addition, once they enter epithelial cells, copper ions are showing an ability to inhibit the activity of the membrane bound Na^+^/K^+^-ATPase [13, 14]. Further, copper is known to induce oxidative stress, olfactory impairment, increased plasma ammonia and disturbed acid-base balance [15, 16, 17]. Acute toxic concentrations of zinc are generally higher than those of copper, yet deleterious effects have been described repeatedly [18, 19]. Unlike copper, zinc uptake is facilitated via a common zinc-calcium transport carrier, located in the mitochondria rich cells of the branchial apparatus [20, 21]. An impaired branchial Ca^2+^-influx and hypocalcemia as an indirect consequence is therefore to be expected [22, 23]. Prolonged elevated zinc uptake will eventually lead to critically high zinc accumulation in tissues where it can generate damaging reactive oxygen species [24]. Cadmium on the other hand is not essential for fishes’ metabolic processes, and is potentially dangerous at lower concentrations compared to the essential metalloids. As it is the case for zinc, cadmium utilizes the Ca^2+^-channel to enter the gills and can affect calcium homeostasis as well [25]. Once accumulated in other tissues, it will induce oxidative stress through the generation of reactive oxygen species at relatively low concentrations [26, 27, 28].

Exposure of freshwater fish to elevated metal levels in the aquatic environment, and the subsequent disturbance in iono- and osmoregulatory processes, typically results in morphological changes of their branchial apparatus [29, 30]. Due to the high plasticity of gill tissue, abovementioned physiological mechanisms can induce tissue damage and/or remodeling. Secondary lamellae of gills serve as a primary site for gas exchange and ion transport in fish, and morphological adaptations either facilitate oxygen uptake or serve as a mechanism for increasing the blood-water barrier [31]. If the presence of the irritant is persistent, different histopathological alterations can occur which could reduce the respiratory surface and impair respiration and physiology of the gills [32]. These histopathological alterations could be quantified to assess adverse effects of specific xenobiotics on the gill tissue, thus giving an insight into the general fish health. Such a methodology is used in a vast number of studies of metals on gill histology, i.e. laboratory exposures [33, 34, 35]; semi-field set-ups [36, 37]; and field studies [38, 39, 40]. A specific pollutant will not necessarily induce pollutant-specific histopathological alterations in gill tissue, but a quantification of the extent and intensity of overall changes enables a comparison between experimental groups in various trials [41, 42, 43]. This method is utilized in the present study, in order to compare the effects of the three metals on the morphology of the branchial apparatus of common carp. Common carp was selected for this exposure assay because of its worldwide distribution, the importance of this species in aquaculture and because it is listed in OECD guidelines as recommended fish species for the testing of chemicals. We selected juveniles, both to prevent gender related influences and because younger life stages are more sensitive to metal exposure than adults.

Exposure concentrations may either be chosen based on the outcomes of environmental studies or come from standard toxicity tests. In the latter case, the toxicity of a substance can be quantified by determining the concentration at which 50% of the exposed individuals die over a time span of 96 hours (96h LC_50_). However, even under controlled conditions the outcomes of these kind of studies are likely to be influenced by confounding factors, such as age of the animals, environmental conditions, and the water chemistry [44, 45, 46]. A query in the EPA ecotox database [47] for 96h LC_50_ values of copper, cadmium and zinc toxicity for common carp, resulted in a wide range of concentrations for each of the metals tested: 96 h LC_50_ for copper between 0.02 and 542 μM [22, 48, 49, 50, 51, 52, 53, 54, 45, 55, 56]; 96 h LC_50_ for cadmium between 1.25 and 159 μM [57, 58, 59, 60, 19, 61]; and 96 h LC_50_ for zinc between 149 and 769 μM [62, 18, 19].

Therefore, an acute exposure experiment was setup first to determine the toxicity and bioaccumulation of copper, cadmium and zinc in common carp under the standardized conditions used in our study. Subsequently, two sublethal equitoxic exposure concentrations were chosen for each metal corresponding to 50% and 10% of the calculated LC_50_-values. The main goal of this second, subacute exposure experiment was to identify the histopathological and physiological effects on the gill tissue of common carp (*Cyprinus carpio*). We hypothesized that we would find metal specific alterations, with copper interfering with sodium transport and inducing more severe gill alterations, and cadmium and zinc primarily interfering with calcium homeostasis and resulting in limited gill damage.

## Materials and methods

### Test animals

Five month old common carp (*Cyprinus carpio)* juveniles were obtained from the fish hatchery at Wageningen University, The Netherlands and kept in reconstituted freshwater made from deionized water (Aqualab, VWR International), supplemented with 4 salts (VWR Chemicals): NaHCO_3_ (1.14 mM), CaSO_4_.2H_2_O (0.35 mM), MgSO_4_.7H_2_O (0.5 mM), KCl (0.05 mM) to reach moderately-hard water (conductivity 308 ± 2.5 μS/cm), as defined by the US Environmental Protection Agency [63]. Each 200L tank was equipped with aeration stones and a biofilter to ensure optimal water quality. The photoperiod was set at 12 hours light, 12 hours dark, and the water temperature was maintained at 20 ± 1.5°C throughout the acclimation period and experiment. A commercial trout feed (Start Premium, 1mm, Coppens) was given *ad libitum* during the acclimation period but the fish were fastened starting 1 day before the trial and continuing throughout the whole experimental period.

### Acute exposure experiment

Exposure tanks consisted of double-walled 10L polypropylene (PP) buckets, each filled with 9 L of EPA medium-hard water and containing 5 fish of 2.63 ± 0.99 g. Two replicate tanks were used per treatment and in each bucket oxygen was provided with an air stone. In order to avoid the accumulation of ammonia and other waste products, 90% of the water was changed daily (semi-static system). To minimize disturbance to the fish, the perforated inner bucket was lifted from the outer bucket and the fish and 1 L of water stayed behind in the inner bucket so that the remaining 8 L of water in the outer bucket could easily be replaced. The pH (8.21 ± 0.03) and water temperature (20 ± 1°C) were monitored daily to ensure stable experimental conditions. The water was spiked with an appropriate volume of the stock solutions of CdCl_2_ (Merck), CuSO_4_·5H_2_O (VWR Chemicals) and ZnCl_2_ (VWR Chemicals), to reach the desired nominal concentrations of copper (0–11 μM), cadmium (0–100 μM) and zinc (0–150 μM) respectively. Water samples were taken right before and after the water renewal, to analyze for the actual metal concentrations. Measured concentrations for each treatment are given in Table 1 and varied for cadmium between 0.00–106.04 μM, for copper between 0.02–10.78 μM, and for zinc between 0.08–129.23 μM.

**Table 1:** Actual metal concentrations measured in the water of the exposure tanks.

Cu (μM)	Cd (μM)	Zn (μM)
0.02 ± 0.01	N.D.	0.08 ± 0.03
0.29 ± 0.03	0.14 ± 0.01	9.97 ± 0.48
0.54 ± 0.02	0.37 ± 0.06	18.11 ± 0.30
0.76 ± 0.06	0.62 ± 0.05	30.23 ± 0.23
1.00 ± 0.08	0.92 ± 0.10	62.47 ± 2.21
1.17 ± 0.09	2.62 ± 0.12	83.85 ± 2.03
2.43 ± 0.06	5.78 ± 0.13	114.60 ± 2.08
4.31 ± 0.05	10.83 ± 0.33	129.23 ± 5.00
6.53 ± 0.29	12.70 ± 0.77	
8.06 ± 0.26	21.20 ± 0.64	
10.78 ± 0.15	44.36 ± 1.41	
	64.79 ± 2.35	
	90.27 ± 2.27	
	106.04 ± 7.14	

The values are represented as mean ± SEM of the 2 replicate tanks, measured over the entire experimental period of 10 days (N.D. Not detectable).

Every three hours for a period of 10 days, the fish were checked for mortality or severe discomfort. Fish were considered in severe discomfort when they remained at the surface, were unable to escape to deeper water when touched or non-responsive to touching at any water depth. When this was the case, fish were considered moribund, most likely not being able to survive the next 3 hour period, and were immediately removed and euthanized using an overdose of neutralized MS222 (Sigma). Generally, moribund fish could easily be detected in copper and zinc exposed fish, but in cadmium exposed fish death often occurred within the 3 hour intermediate period without earlier signs of severe discomfort. Dead and euthanized moribund fish were stored at -20°C for later whole-body metal analysis. After 10 days of exposure, the experiment was terminated and all remaining fish were collected, immediately euthanized with an overdose of neutralized MS222 (Sigma) and stored at -20°C for whole-body metal analysis. In total 330 fish were used of which 92 survived until the end of the experiment without any severe signs of distress, after which they were euthanized as described above. Of the remaining fish, the first 15 exposures (150 fish, 50 per metal) to concentrations based on literature data showed very quick mortality, with a 100% mortality mostly reached within 2 days, too fast to detect moribund fish before death. Of the remaining 88 fish, 31 were euthanized during the course of the experiment as they were considered in severe distress, and 57 were found dead, again, usually in the cadmium exposures.

### Sublethal metal exposures

In a similar set-up (semi-static system), fish from the same batch, now weighing 15.74 ± 6.67 g were stocked in the 10L PP buckets and held in an acclimated room, with a starting density of 6 fish per bucket and 3 replicate buckets per treatment. The pH and temperature was monitored daily with a field meter (Hach HQ30d) and water was renewed daily as described above. It was chosen to expose the fish to control conditions and a low and a high sublethal concentration of each metal, respectively 10% and 50% of the 96h LC_50_ obtained from the acute exposure experiment. Nominal concentrations were for Cd 0.00, 0.02 and 0.10 μM, for Cu 0.00, 0.08 and 0.40 μM, and for Zn 0, 3 and 15 μM. Each exposure condition was executed in triplicate and lasted for 7 days. On day 1, 3 and 7, six fish were sampled from each exposure group (2 per bucket) for metal and electrolyte analysis. The fish were euthanized with an overdose of neutralized MS222 (Sigma) and thoroughly rinsed before dissection of the gills. In the final sampling, the second gill arch from the left side was collected for histological analysis.

### Determination of metal- and electrolyte content in tissue

For the acute exposure experiment, whole bodies were acid digested, whereas for the subacute exposure experiment the gill arches were collected for metal analysis. The samples were dried at 60°C for 48 hours. They were set to cool down in a desiccator before the dry weight was taken on a precision balance (Mettler AT261 DeltaRange). Next, trace-metal-grade HNO_3_ (69%, Seastar Chemicals) was added to the samples, blanks and standard reference material (SRM-2976, Mussel tissue, National Institute of Standards and Technology), and left to settle after which H_2_O_2_ (29%, Seastar Chemicals) was added. Volumes for the digestions were adjusted according to tissue weight, for the carcasses (5ml HNO_3_ followed by 0.5ml of H_2_O_2_) and the gills (1.5ml of HNO_3_ followed 0.05 ml of H_2_O_2_). The digestion process was initiated at room temperature for 12 hours, followed by a 30 minute-incubation in a hot block (Environmental Express) at 115°C. The total metal concentration and major ion levels (Na, K, Mg, and Ca) in the digested tissues and water samples were determined with a quadrupole inductively coupled plasma mass spectrometer (ICP-MS; iCAP 6000 series, Thermo Scientific). The calculated recoveries of the reference material were 102.67, 94.62 and 100.07% for cadmium, copper and zinc respectively. The recoveries for the major ions K, Mg, Na and Ca were 98.06, 89.65, 92.89 and 94.41 respectively.

### Histology of the gills

The left second gill arch of control and 7-day exposed fish was collected for histological analysis and placed in 4% aqueous formaldehyde (Sigma). The fixation period lasted for 48 hours, before the gills were transferred to 70% ethanol. Samples were later dehydrated in a graded ethanol series with an automatic tissue processor (Leica TP 1020), cleared in xylene, embedded in paraffin and subsequently sectioned transversely at nominal thickness of 5 μm. Slides were stained with a linear stainer (Leica ST4040) using hematoxylin and eosin (H/E) [64]. Micrographs presented in the paper were taken with a Leica DM LB microscope equipped with a Leica DFC 295 camera.

### Semi-quantitative scoring system

For the assessment of the intensity and extent of histopathological alterations in gills, a semi-quantitative scoring system developed [41] was used. Each slide was blinded and evaluated for the extent of histopathological alterations by assigning the following score value: 0—no alteration present; 2—mild occurrence, 4—moderate occurrence and 6—severe occurrence. Intermediate values were not used, and consensual agreement between two histopathologists was done if ambiguity was present. The list of alterations found in the present study is shown in the results section (Table 4), and each alteration is marked with the importance factor (w), ranging from 1 (minimal alteration) to 3 (alteration of marked importance), according to [41]. Moreover, each alteration is classified in one of the following reaction patterns: circulatory (telangiectasis, hyperemia, oedema of primary epithelium and secondary epithelium), regressive (structural and architectural alterations, goblet cells in secondary lamellae, necrosis), progressive (hypertrophy, hyperplasia of epithelium and goblet cells), inflammatory (infiltration of leukocytes) and neoplastic changes (not found in the present study). For quantification of histopathological alterations, and obtaining a reaction index and a total gill index, following formulas were used:

- (a) Reaction index of gills (I_rp_)
  $$I_{rp}=\sum_{alt}\left(a_{rp\;alt}\times w_{rp\;alt}\right)$$ (Eq 1)
- (b) Histopathological index of the gills:
  $$I_{G}=\sum_{rp}\sum_{alt}\left(a_{rp\;alt}\times w_{rp\;alt}\right)$$ (Eq 2)
  where *I*_*G*_ represents the gills index, *rp*- reaction pattern, *alt*- the alteration, *a*- score value, and *w*- importance factor.

### Statistics

Data are reported as mean ± standard error of the mean (SEM). GraphPad Prism 7.00 (GraphPad Software) was used to calculate the incipient lethal level (ILL) with the provided one-phase decay equation [65]. All other data analysis was done with the open source software package R (version 3.4.0), and graphs were made with the *ggplot2 package* [66]. The normality of the data and homogeneity of the variances was tested with the Shapiro-Wilks and Levene’s test, respectively. Parametric datasets were analyzed with ANOVA, followed by Tukey HSD test to make pairwise comparisons of significant differences. If the assumptions for parametric tests were not met, a Kruskal-Wallis and Dunn test were executed on the dataset. Linear regressions were calculated using Pearsons correlation. The dose-response curve (drc) package [67] was used to fit a four-parameter logistic model (Eq 3) to the mortality dataset obtained from the acute toxicity test. A lack-of-fit test was executed to evaluate the acceptability of the model. Subsequently, the parameter estimates, including the LC_50_-values, and their corresponding standard deviations and p-values could be extracted from the model.

$$f=(x,(b,c,d,e))=c+\frac{d-c}{1+exp\{b(\log\left(x\right)-\log\left(e\right))\}}$$ (Eq 3)

where “e” represents the LC_50_, “b” is the slope around e, and c and d are the respectively the lower and upper limit.

### Compliance with ethical standards

The fish husbandry and experiments complied with the regulation of the Federation of European Laboratory Animal Science Associations and were approved by the local ethics committee, University of Antwerp (Permit Number: 2015–94 Project 32252). VD, LB, RB and GDB all obtained the certificate of Experimenter Category C as defined in Art 11§4 and annex 8 of the Royal Decree of April 6, 2010 concerning the protection of laboratory animals. BR (gill microscopy) and MSS (metal and electrolyte analysis) analyzed samples after the exposures were done.

## Results

### Acute metal exposure

**Fig 1:**
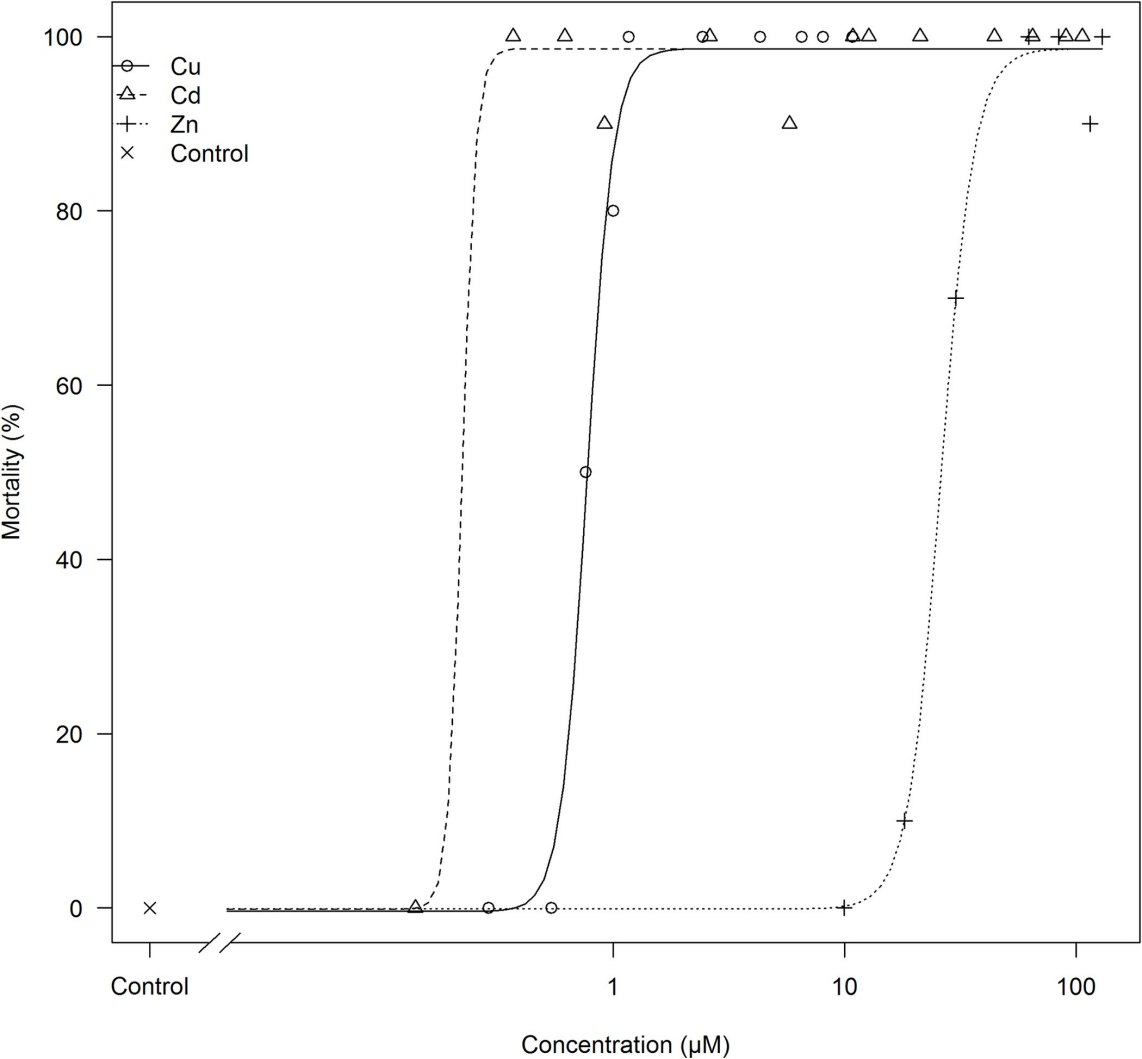
The four-parameter logistic fit of the model to the data set of copper, cadmium and zinc exposure for 240 hours. Mortality is plotted as a function of the nominal concentration of the respective metal ion (in μM).

The exposure concentrations initially selected based on literature data resulted in high mortalities within the first 4 days of exposure. Therefore, additional tests were set up at lower concentrations to gather reliable toxicity data, which did end up in 11 tested concentrations for copper, 14 for cadmium and 8 for zinc. Measured concentrations for each treatment are given in Table 1 and varied for cadmium between 0.00–106.04 μM, for copper between 0.02–10.78 μM, and for zinc between 0.08–129.23 μM. The copper and zinc tests induced clear dose-dependent mortality, where the higher concentrations caused 100% mortality for all the fish, whereas the tanks spiked with lower doses of the metals, were characterized by intermediate to zero mortality (Fig 1). In contrast, 12 out of the 14 tested concentrations for cadmium caused 90–100% mortality in the tanks, whereas the lowest 2 concentrations did not result in any dead fish over the entire experimental period. Typically, moribund fish could easily be detected in copper and zinc exposed fish after which they were euthanized as described above, but in cadmium exposed fish death often occurred sudden, without earlier signs of severe discomfort. The fit of the four-parameter logistic model to the mortality data is presented in Fig 1, and the lack-of–fit test resulted in a p-value of 0.670 which indicates that the model is reliable to describe the dose-response relationships. Table 2 summarizes the values of the parameters describing the individual dose-response curves of the three metals, assuming both 96h- and 240h exposure scenarios. When tested over an experimental period of 10 days, zinc can be considered the least toxic metal of this study. Instead, waterborne copper and cadmium are 100-times more lethal towards common carp juveniles. The 96h LC_50_ values were calculated based on the four-parameter logistic model as well and resulted in 0.20±0.16 μM (p = 0.22) for cadmium, 0.77±0.03 μM (p = 2.20e^-16^) for copper, and 29.89±9.03 μM (p = 1.40e^-03^) for zinc.

**Table 2:** Parameters used for the logistic function to plot the dose-response relationship for the 96h and 240h exposure experiment.

Element	Duration	b	c	d	e
Cu	96h	-7.99	-0.37	97.16	0.76
	240h	-7.69	-0.40	98.62	0.77
Cd	96h	-1.004	-55.36	97.16	0.20
	240h	-14.64	-0.17	98.62	0.22
Zn	96h	-1.80	-22.20	97.16	29.89
	240h	-6.03	-0.10	98.62	26.03

“e” represents the LC50, “b” is the slope around e, and c and d are the respectively the lower and upper limit.

**Fig 2:**
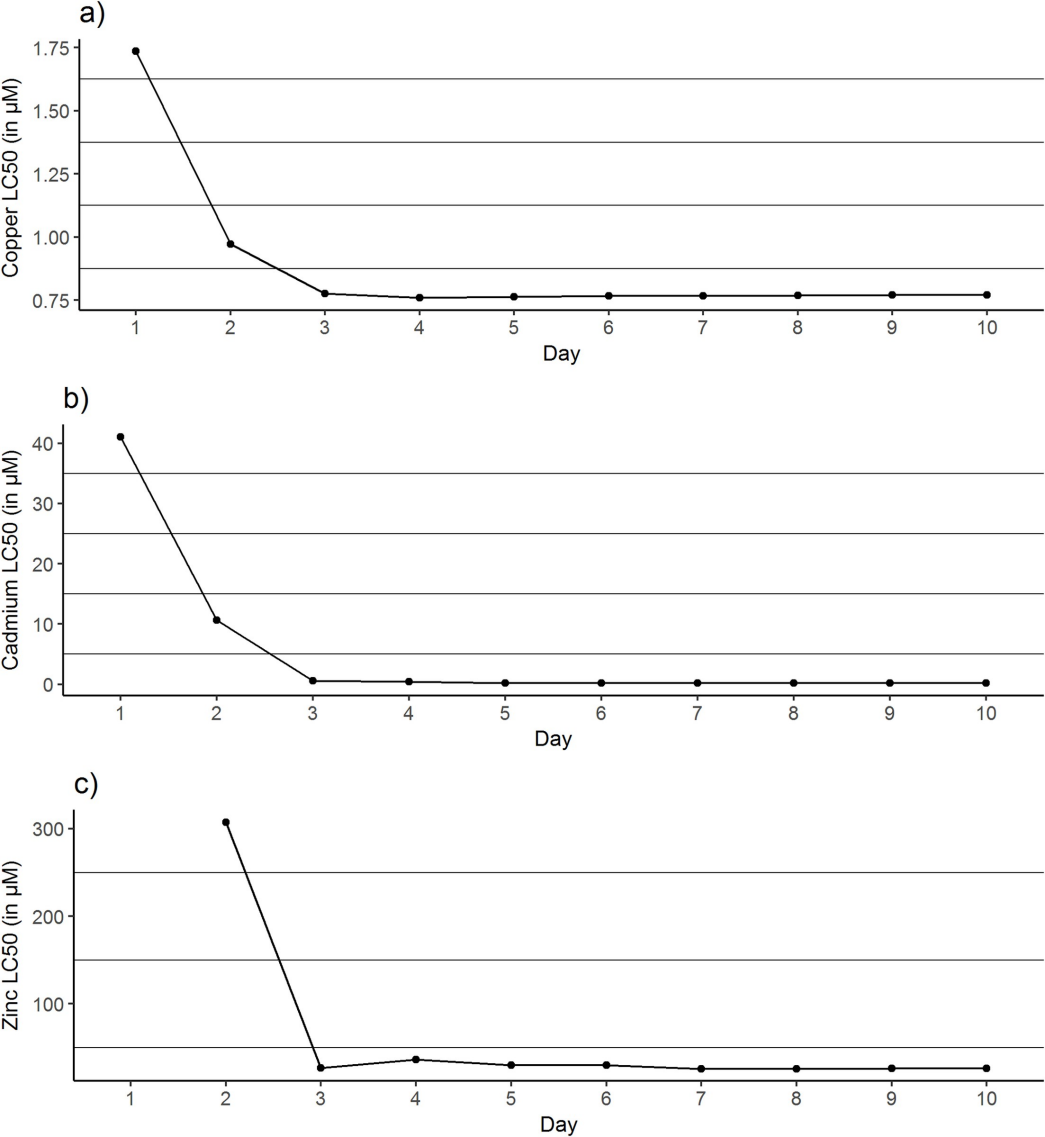
The trend in lethal level for 50% of common carp juveniles ((LC_50_) for the three metals copper (a), cadmium (b) and zinc (c) tested over a time span of 10 days (240 hours).

Fig 2 shows the trend in lethal concentration of the three metals over time. The asymptotes of each curve are approaching the concentrations below which 50% of the fish will live indefinitely, i.e. the incipient lethal level (ILL). In case of a copper exposure (Fig 2A) this level will be reached after 3 days and for cadmium (Fig 2B) and zinc (Fig 2C) after 5 and 6 days of exposure, respectively. The ILL for copper, cadmium and zinc calculated in this experiment are respectively 0.77 μM, 0.16 μM and 28.33 μM.

In a first analysis, a comparison was made between the metal content in the survivors and the non-survivors of the exposure experiment. The respective values for cadmium, copper and zinc were corrected for background metal content of the control fish, in order to get a better picture of actual metal accumulation due to the exposure. In the copper exposure experiment the survivors accumulated on average 0.20 ± 0.01 μmol/g DW and the fish that died 0.17 ± 0.01 μmol/g DW. For the zinc exposures a metal content of 1.30 ± 0.27 μmol/g DW and 1.05 ± 0.27 μmol/g DW was measured in the survivors and dead fish, respectively. Neither for the copper exposure, nor for the zinc exposure these differences between survivors and victims were found to be significantly different. In contrast, there was a significantly (p = 0.001) higher cadmium content measured in the fish which died after the cadmium exposure (0.26 ± 0.03 μmol/g DW) compared to the survivors (0.05 ± 0.02 μmol/g DW).

**Fig 3:**
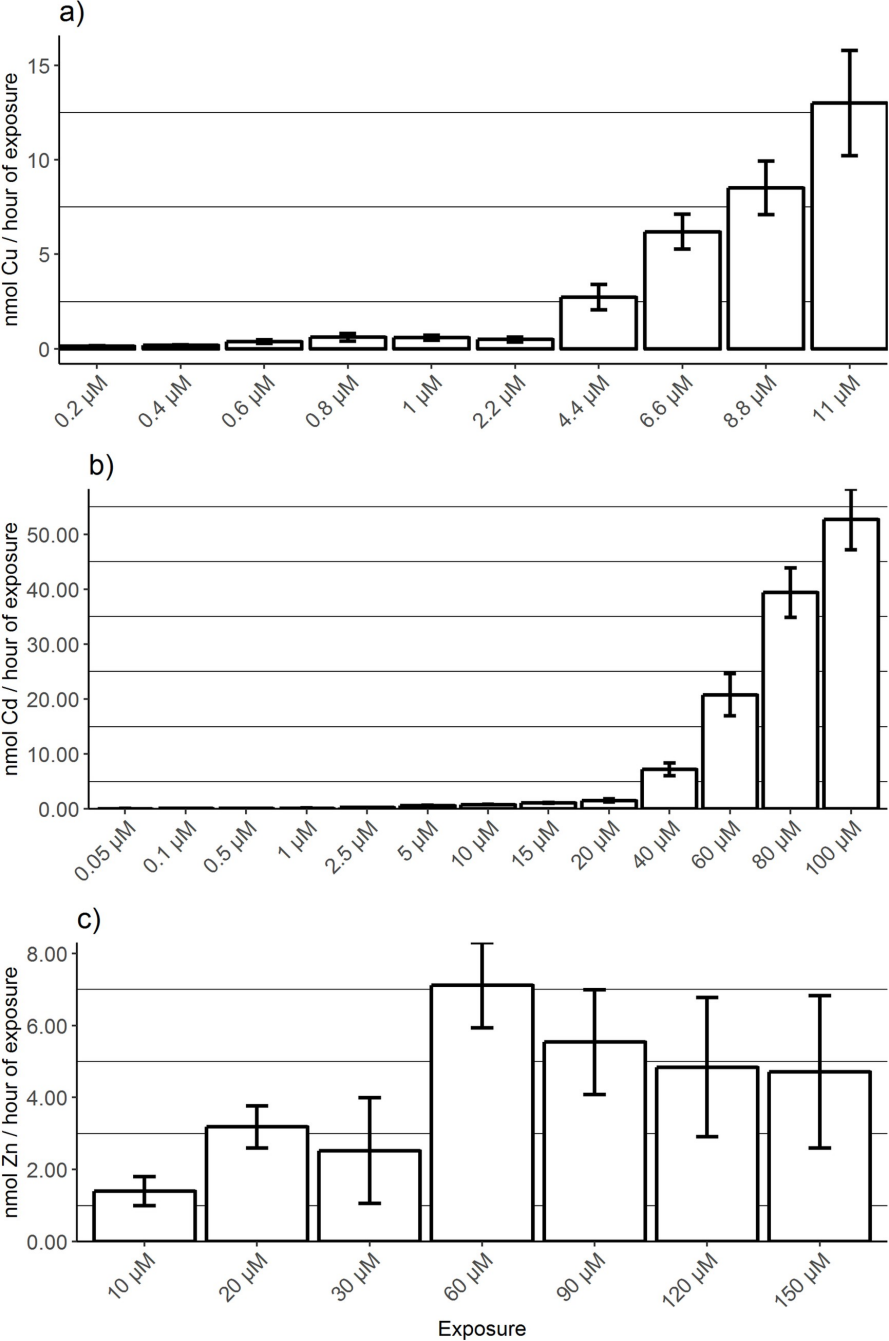
Metal uptake rate (mean ± SEM) during the 240 hours toxicity screening test for the different exposure concentrations (in μM), for copper (a), cadmium (b) and zinc (c) exposure. The total metal content (in μg/g dry weight) measured in the whole body is corrected for the average value of the respective metal in the control fish. The y-axis expresses the uptake rate in hours of exposure with error bars as standard error of mean.

In a second analysis a concentration dependent uptake rate was calculated, where the accumulated metal content was corrected for the duration of exposure which obviously differed depending on the time of mortality. For copper exposures, copper uptake rate is very low and similar (0.0002–0.0008 μmol/g DW/h) for the 6 lowest exposure concentrations (0.2–2.2 μM). Starting from an exposure dose of 4.4 μM copper, the uptake rate starts to increase, with the highest rates observed for the fish exposed to 11 μM copper (Fig 3A). The fish exposed to different concentrations of cadmium showed a similar pattern in uptake with a minimal increase (0.00–0.002 μmol/g DW/h) for the 9 lowest concentrations, and a notable rise in uptake rate starting from an exposure concentration of 40 μM (Fig 3B). The fish exposed to zinc did not show such a concentration dependent increase in uptake rates (Fig 3C). All calculated uptake rates were within the same range (0.10–0.52 μg/g DW/h), except for the fish exposed to 10 μM (0.002 μmol/g DW/h) and 20 μM (0.004 μmol/g DW/h) of zinc.

### Sublethal metal exposure

In the sublethal metal exposure, we aimed at using equitoxic concentrations. As the toxicity of the metals in absolute concentrations differed substantially, we exposed the fish to the same toxic units, 10 and 50% of the 96h LC_50_ determined above. The actual concentrations measured in the water samples were for cadmium below detection limit for control, and for exposures 0.018 ± 0.000 and 0.083 ± 0.002 μM, for copper 0.013±0.002 for control, and 0.074 ± 0.002 and 0.333 ± 0.007 μM for exposures, and for zinc 1.477±0.714 for control, and 2.547 ± 0.052 and 10.765 ± 0.570 μM for exposures. We chose to use fish from the same batch, with the same genetic background. As this experiment was done a few months after the acute exposure, fish were larger in size. Size affects toxicity, but all fish were still juveniles as common carp only mature at 2–3 years of age. Therefore absolute toxicity values might have shifted to slightly higher values, but this would have happened proportionally for all metals leaving the main assumption of exposing the fish to equal toxic units valid.

**Fig 4:**
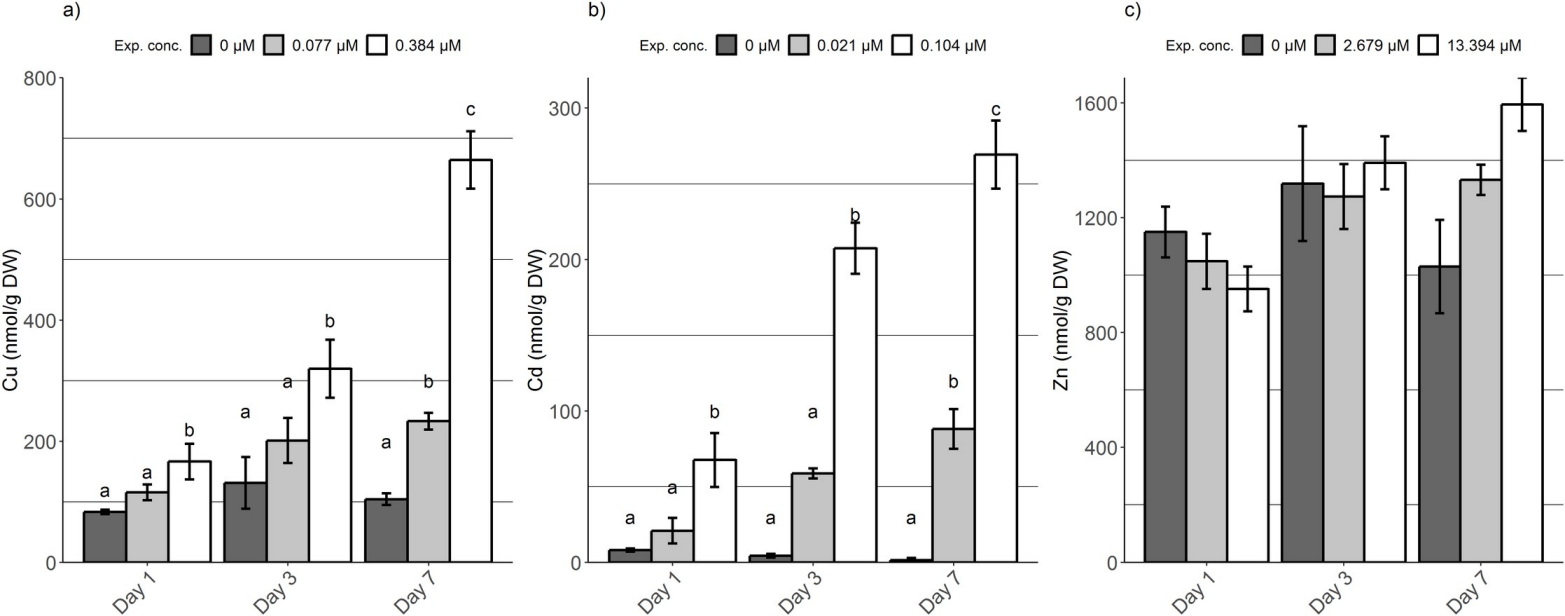
Total copper (a), cadmium (b) and zinc (c) accumulated in the gills expressed in micrograms per gram of dry weight (mean ± SEM). The metal content was measured after 1, 3 and 7 days of exposure for the 3 experimental groups; control (dark grey), low dose (light grey) and high dose (white). Day 0 refers to the time zero control group (black). Letters denote significant differences among treatments and within 1 sampling day, found with Dunn’s test of multiple comparisons (Bonferroni adjusted p-value rejects H_0_ if p< = 0.05/2).

At the start of the experiment cadmium was not detectable in the fish gills, whereas the copper and zinc content was 0.08 ± 0.006 μmol/g and 16.45 ± 1.19 μmol/g dry weight respectively. A time- and concentration- dependent increase in copper accumulation in the gill tissues was seen as a result of waterborne copper exposure (Fig 4A). At day 1 and 3 only the fish exposed to the high copper concentration (50% of 96h LC_50_) accumulated significantly higher levels of copper than the control fish (chi-squared = 6.5, df = 2, p = 0.02). After 7 days, both groups exposed to the low and high copper concentration had significantly more copper in their gill tissue compared to the control group, 0.23 ± 0.01 μmol/g and 0.66 ± 0.05 μmol/g, respectively (chi-squared = 25.3, df = 2, p = 0.001). The net uptake of copper was estimated by correcting the measured level with the average background level in the gills of the control fish. Such a calculation shows that the fish exposed to the low and high dose of copper accumulated approximately 0.13 and 0.56 μmol copper. This corresponds respectively to a 123 and 538% increase in gill copper levels after 7 days of exposure and a net uptake rate of 0.0008 and 0.003 μg/g DW/h respectively.

Cadmium levels in the gills did also increase in a time- and concentration- dependent manner (Fig 4B). At day 1, the fish exposed to the high dose of cadmium did accumulate significantly more cadmium than the control fish, and this remained the case for the entire experiment (chi-squared = 10.4, df = 2, p = 0.01). A similar pattern was seen for the treatment group exposed to a low dose of cadmium, however statistically significant higher levels of cadmium in the gill tissue compared to the control group were only observed after 7 days of exposure (chi-squared = 31.0, df = 2, p = 0.01). At the end of the experiment, low- and high dose treated groups accumulated on average 0.09 ± 0.01 and 0.27 ± 0.02 μmol cadmium per gram of dry gill tissue which corresponds to a net uptake rate of 0.0005 and 0.001 μmol/g DW/h respectively.

The variation in zinc content of the gills of the carp juveniles was rather high during the entire experiment (Fig 4C), also in the control fish. No significant differences in zinc accumulation could be observed as a result of the treatment. It is only at day 7 that the expected trend started to take shape with small, but dose dependent differences in zinc accumulation among treatment groups. The fish exposed to the lowest and highest dose had respectively 20.36 ± 0.81 and 24.39 ± 1.42 μmol/g of zinc in their gill tissue on the last sampling day. When corrected for the zinc content in the control fish, this resulted in a net accumulation of 4.61 and 8.63 μmol zinc or a 30 and 54% increase, respectively. This corresponds to an uptake rate of 0.03 and 0.05 μg/g DW/h respectively.

A linear regression model was used to fit the specific gill electrolyte levels (Na, K, Mg and Ca) to the accumulated metals (Table 3). Copper was the only metal which induced a significant change on the electrolyte content in the gill tissue. On day 1 a transient slightly positive correlation between accumulated copper and K and Mg levels was observed (resp., R^2^ = 0.27, p = 0.02 and R^2^ = 0.22, p = 0.03). Gill sodium levels were clearly affected by copper content. Starting from day 3, lower sodium contents were significantly associated with increasing copper concentrations (R^2^ = 0.27, p = 0.02). This trend continues on day 7 where a highly significant correlation between sodium and copper was observed (R^2^ = 0.8, p = 2.96e^-7^). Neither the zinc-exposure, nor the cadmium exposure did reveal any significant correlation between accumulated metals and gill electrolyte levels.

**Table 3:** The results of the linear regression analysis to find associations between accumulated metals in the gills and electrolyte levels. The slope and the Pearson correlation coefficient are provided with the corresponding p-value. Significant associations (p < 0.05) are highlighted in with “*”, N = 18.

		Cu-exposure			Cd-exposure			Zn-exposure		
		Slope	Adj R^2^	p-value	Slope	Adj R^2^	p-value	Slope	Adj R^2^	p-value
**Na**	**Day 1**	-34.12	-0.05	0.67	-44.34	-0.04	0.5	0.64	-0.03	0.49
	**Day 3**	-118.59	0.27	0.02*	-30.84	0.07	0.17	-0.73	-0.04	0.58
	**Day 7**	-107.5	0.8	2.96e-07*	-31.13	0.09	0.16	-0.75	-0.01	0.38
**K**	**Day 1**	429.02	0.27	0.03*	-83.07	-0.04	0.53	-1.00	-0.05	0.68
	**Day 3**	-41.45	-0.05	0.59	-26.28	-0.04	0.53	-1.54	-0.04	0.54
	**Day 7**	-40.03	0.01	0.3	0.80	-0.09	0.98	-0.95	-0.04	0.59
**Mg**	**Day 1**	38.57	0.22	0.03*	3.41	-0.06	0.74	0.02	-0.06	0.88
	**Day 3**	-3.61	-0.04	0.52	-5.20	0.1	0.14	0.01	-0.07	0.94
	**Day 7**	1.41	-0.04	0.66	-3.70	-0.05	0.53	0.09	-0.04	0.6
**Ca**	**Day 1**	773.17	0.15	0.07	242.75	0	0.34	2.45	-0.05	0.64
	**Day 3**	-45.70	-0.06	0.74	7.23	-0.08	0.93	-4.85	0.05	0.2
	**Day 7**	-46.93	-0.04	0.55	-58.75	-0.08	0.74	2.94	-0.04	0.54

**Fig 5:**
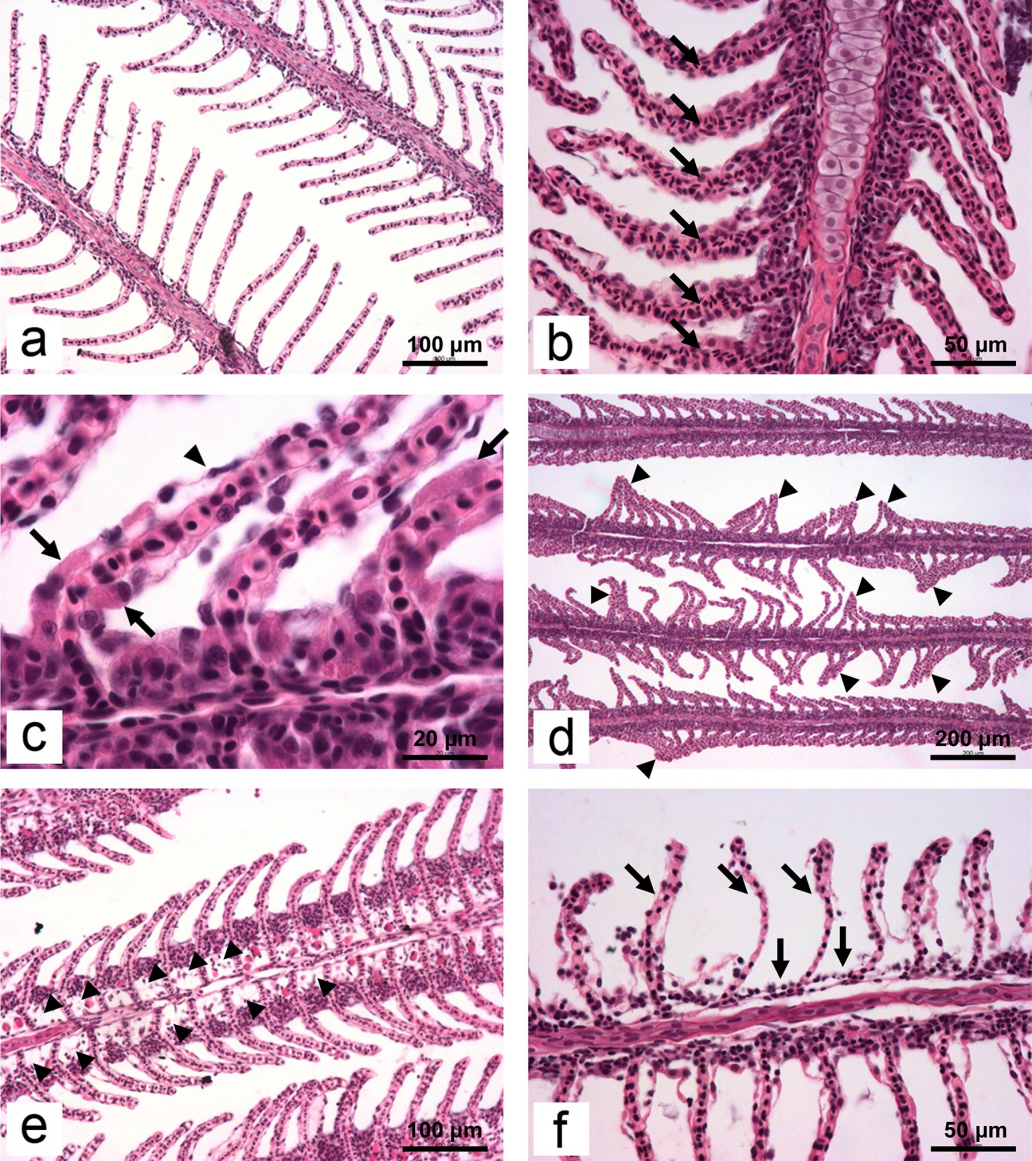
Micrographs of some of histopathological alterations found in the gills of common carp in the present study: a) normal tissue structure (H/E x200); b) note the extensive hyperemia on secondary lamellae (arrows; H/E x400); c) hypertrophy of squamous epithelial cells (arrows); compare thickness of these cells to non-pathological cell (arrowhead). Furthermore, note the size of the nuclei in both type of cells (H/E x1000); d) disturbed architecture of secondary lamellae: curling and fusion (arrowheads; H/E x100); e) oedema of primary filament (arrowheads; H/E x200); f) total rupture of epithelium leading to necrosis (arrows; H/E x400).

Histopathological alterations found in gills of common carp (Table 4) were generally mild at these sublethal exposure levels and no dose-dependent relationship could be established using semiquantitative scoring. At the end of experiment, histopathological changes in the control group were either totally absent or some of them were rarely noticed (Fig 5A). Moderate scores were present only in alterations with low histopathological significance according to importance factor. They include hyperemia (Fig 5B) in fish from Cu_10_ and Cd_10_ groups, hypertrophy (Fig 5C) in Cu_50_ group, structural and architectural alterations (Fig 5D) in Cd_50_ group, and oedema of primary epithelium (Fig 5E) in Cd_50_, Zn_10_ and Zn_50_ groups. As we defined moderate scores ranging from 2 to 4 in the exposed carp, the majority of them were significantly higher compared to the control group. Gill necrosis, as an irreversible alteration with highest importance factor, was present only in small scattered areas. (Fig 5F).

**Table 4:** Mean values of histopathological scores of common carp from experimental groups.

	RP	IF	Con	Cu_10_	Cu_50_	Cd_10_	Cd_50_	Zn_10_	Zn_50_
Telangiectasis	C	1	0.0 ± 0.0	0.3 ± 0.8	0.3 ± 0.8	0.0 ± 0.0	0.0 ± 0.0	0.0 ± 0.0	0.3 ± 0.8
Hyperemia	C	1	0.7 ± 1.0^a^	4.0 ± 1.8^b^	0.3 ± 0.8^ab^	3.3 ± 1.6^b^	2.7 ± 2.7^ab^	1.7 ± 0.8^ab^	1.7 ± 0.8^ab^
Oedema of primary epithelium	C	1	0.0 ± 0.0^a^	0.3 ± 0.8^a^	0.0 ± 0.0^a^	1.3 ± 1.6^ab^	4.0 ± 1.8^b^	2.7 ± 1.0^b^	2.3 ± 2.0^b^
Oedema of secondary epithelium	C	1	1.0 ± 1.1	2.0 ± 2.2	3.7 ± 2.3	1.7 ± 0.8	3.7 ± 2.0	2.7 ± 1.0	1.7 ± 1.5
Hypertrophy	P	1	0.0 ± 0.0^a^	1.3 ± 1.0^ab^	2.7 ± 1.6^b^	0.3 ± 0.8^a^	0.0 ± 0.0^a^	1.0 ± 1.1^ab^	0.7 ± 1.0^a^
Hyperplasia of epithelium	P	2	0.3 ± 0.8	1.7 ± 1.5	0.7 ± 1.6	0.7 ± 1.0	0.7 ± 1.0	0.7 ± 1.0	0.3 ± 0.8
Hyperplasia of goblet cells	P	2	1.0 ± 1.1	1.0 ± 1.7	1.7 ± 1.5	0.0 ± 0.0	1.0 ± 1.1	0.0 ± 0.0	0.3 ± 0.8
Struct. and architect. alterations	R	1	0.7 ± 1.0^a^	2.3 ± 2.3^ab^	2.7 ± 1.0^ab^	2.3 ± 0.8^ab^	3.3 ± 1.6^b^	1.7 ± 0.8^ab^	1.7 ± 0.8^ab^
Goblet cells in sec. lamellae	R	1	0.3 ± 0.8	0.0 ± 0.0	0.7 ± 1.0	0.0 ± 0.0	0.0 ± 0.0	0.0 ± 0.0	0.0 ± 0.0
Necrosis	R	3	0.0 ± 0.0	0.3 ± 0.8	0.3 ± 0.8	0.0 ± 0.0	0.7 ± 1.0	0.0 ± 0.0	0.3 ± 0.8
Infiltration of leukocytes	I	2	0.0 ± 0.0	1.0 ± 1.7	0.0 ± 0.0	1.7 ± 2.0	1.0 ± 1.7	0.7 ± 1.0	0.0 ± 0.0
Gill index circulatory changes	C	-	0.7 ± 1.0^a^	4.7 ± 2.4^ab^	0.7 ± 1.0^a^	4.7 ± 1.0^ab^	6.7 ± 3.7^b^	4.3 ± 1.5^ab^	4.3 ± 1.5^ab^
Gill index progressive changes	P	-	1.7 ± 2.3	5.7 ± 5.0	5.7 ± 4.8	1.7 ± 2.7	2.3 ± 2.3	2.3 ± 2.3	1.7 ± 2.3
Gill index regressive changes	R	-	2.0 ± 1.8^a^	5.3 ± 3.9^ab^	8.0 ± 2.5^b^	4.0 ± 1.3^ab^	9.0 ± 3.5^b^	4.3 ± 1.5^ab^	4.3 ± 2.3^ab^
Gill index inflammatory changes	I	-	0.0 ± 0.0	2.0 ± 3.3	0.0 ± 0.0	3.3 ± 3.9	2.0 ± 3.3	1.3 ± 2.1	0.0 ± 0.0
Gill total histopathological index	-	-	4.3 ± 2.9^a^	17.7 ± 10.8^b^	14.3 ± 5.0^ab^	13.7 ± 5.6^ab^	20.0 ± 11.7^b^	12.3 ± 4.5^ab^	10.3 ± 2.9^ab^

Scores are presented as mean values ± SEM; tissue alterations were scored as follows: 0 = none, 2 = mild, 4 = moderate and 6 = severe; mean values followed by different superscript letters in the same row were significantly different (either one-way ANOVA, followed by Tukey`s HSD test or Kruskal-Wallis test, in both cases p < 0.05); IF—importance factor; RP—reaction pattern: C—circulatory changes, P—progressive changes, R—regressive changes, I—inflammatory changes.

Calculated HP indices of different reaction patterns observed after 7 days of exposure are presented in Table 4. As with the individual alterations, the control group showed lowest values in all indices. In the HP index of progressive changes (I_GP_) and in the HP index of inflammatory changes (I_GI_), no statistical significance was established between groups. In the HP index of circulatory changes (I_GC_), the Cd_50_ group was higher compared to both control and Cu_50_ group (p<0.05), while in the HP index of regressive changes (I_GR_), fish exposed to highest concentrations of copper and cadmium had elevated score values compared to the control (P<0.05). The cumulative index of all histopathological alterations, I_GT_, showed that fish in the Cu_10_ and Cd_50_ groups underwent the highest impact compared to fish from control group (p<0.05).

## Discussion

The acute exposure experiment indicated a much higher toxicity of copper and cadmium, compared to what was expected based on the literature review. Several explanations for this outcome can be given. First of all, the size and age of the fish will greatly influence the tolerance of fish towards a pollutant [68]. [54], conducted a similar experiment with copper-exposed common carp and found a 96h LC_50_-value of 10.4 μM. The carp used for that experiment had an average weight of 60 g compared to the 2.6 g juveniles used for this experiment, which most likely explains the over 10-fold higher tolerance to waterborne copper (LC_50_ 96h: 0.77±0.03 μM). For cadmium and zinc higher lethal levels were also reported, which can similarly be attributed to the fact that larger fish were used [69, 61]. Secondly, the water chemistry does also play a major role in metal availability, and thus toxicity. Calcium and sodium ions are typically decreasing the toxicity of metals at the biotic ligand, the fish gills, and are also known for reducing the levels of histopathological alterations when fish are exposed to metals in the hard water [70, 71, 72]. Calcium is also known for its protective role, due to the change in gill permeability or competition with other ions for the binding sites [73, 74]. The use of medium-hard water, in which fish were exposed during the present study, could explain the lower number of histopathological alterations and the generally mild intensity of changes compared to other studies. Not all researchers do consistently report the chemical characteristics of the exposure water, which makes it hard to relate differences in experimental outcome to this aspect. A study [18] does provide detailed information on the water chemistry. The carp were comparable in size to the fish used here, but the obtained LC_50_-value of zinc was approximately 5 times higher (149 μM) than in our experiment. This higher tolerance towards zinc found by these researchers could be related to the nearly 6-fold higher concentration of Ca^2+^-ions in their exposure tanks [18] compared to the Ca^2+^ levels in our reconstituted water. Further, physical parameters like pH and temperature are also known to influence the bioavailability and uptake rate of metals, which might contribute to differences in lethal concentrations with previous studies [75, 76].

In toxicology, a 96-hour exposure experiment is the most common procedure to evaluate the toxicity of a particular compound in fish, since it is obligate and required in the EU for regulatory testing and reporting [77]. Yet, this time span has been debated within the scientific community, as some studies show that the concentration below which 50% of the fish will survive, cannot be determined after only 4 days of exposure [78, 54]. Our study showed that the time to reach the ILL is pollutant-dependent. The results suggest that in our copper experiment, the classic 4-day exposure period was long enough to provide reliable toxicity data. However, incipient lethal levels of cadmium and zinc were only reached after 5 and 6 days of exposure, which advocates for longer exposure experiments.

The total metal accumulation was quantified both in the whole body, in the first experiment, and in the gills in the subacute exposures. In general, a clear increase in copper and cadmium content, proportional to the treatment dose was observed. Zinc, however, did not show such a clear dose-dependent increase. It is known that calcium can competitively inhibit the uptake of zinc [79]. However, this is even more true for cadmium, where the dose-dependent uptake remained obvious. More likely, the naturally high zinc content in the gill tissue (16.45 μmol/g DW) demands a longer exposure period to discern between control and treatment groups. Finally, the fact that zinc is an essential metal implies that fish are able to control the overall uptake of this metal respective to their own needs. Several transmembrane proteins responsible for the import of zinc, have been identified for fish and were found to have a different affinity for waterborne zinc [80]. Previous studies suggest that the activity of these high-affinity zinc importers can be regulated as a consequence of environmental zinc concentrations [81, 82] so this might explain the slow increase in zinc content in the gills. As for zinc, copper is also essential for basic metabolic processes in animals. Therefore, similar regulatory mechanisms are to be expected to maintain copper viable levels in the cells. Clearly, carp were less able to control copper accumulation in comparison to zinc accumulation at equitoxic concentrations. Three uptake pathways for copper have been suggested in fresh water fish; the apical Na^+^-channel, a divalent cation transporter (DMT1) and a high affinity copper transporter (Ctr1) [10, 83, 11]. From a toxicological point of view clear copper accumulation could be attributed to the failure of regulating one or some of the above-mentioned uptake pathways.

When exposed to copper, we found some small scattered areas of necrosis as reported before [84, 85]. Partial lamellar fusion in the form of fused tips of secondary lamellae was more common. Partial or even complete lamellar fusion of lamellae has been detected before under copper exposure, however mostly at much higher copper concentrations [86, 87, 88, 89, 90], and could compromise respiration. Hypertrophy of secondary lamellae and their tips occurred in the Cu_50_ group in a very similar way to what was seen in common carp exposed to 1.72 μM copper [15]. The Cu_50_ group showed the highest level of semiquantitative scores. However, the Cu_10_ group displayed higher levels of hyperemia, a consequence of an increase in blood flow caused by vasodilation of gill arteries, leading to a higher gill index. This physiological adaptation is characteristic for the second phase of the stress response [91] and could point out to an adaptation phase in fish exposed to lower levels of copper. It is widely accepted that copper has a damage-repair mode of action and that deleterious effects are the most pronounced from 4 to 24 h following exposure [14, 15] after which gills start to recover, although full recovery is possible only in copper-free water [84]. These histological changes in the fish gills due to copper exposure are mainly explained by the disruptive physiological effect of the element on the overall ion balance. We observed a reduced sodium level in the gills, similar to other studies which have seen a drop in sodium and chloride levels in the plasma, concomitant with an increase in potassium levels. This effect was seen in a number of freshwater fish, such as common carp, Nile tilapia (*Oreochromis niloticus*) and *Prochilodus scrofa* [15, 92]. The main explanation of this sodium effect lies within shared use of sodium and copper ions of the apical sodium channel causing a competitive inhibition of sodium uptake [10] and the negative correlation between the level of copper in gills and Na^+^/K^+^- ATPase activity [14, 92]. Hyperplasia of mitochondria rich cells, rich in Na^+^/K^+^- ATPase, as well as mucous cells occurs as a compensatory mechanism for the loss of ion uptake capacity [70, 10, 93]

Although cadmium has repetitively demonstrated to inhibit ion-transporting enzymes like Ca^2+^-ATPase and Na^+^/K^+^-ATPase [70, 94, 95] with additional competition of cadmium with Ca and K at the apical calcium channel [96], our study did not indicate any loss of these ions after an exposure to 0.021 or 0.104 μM cadmium. Previous studies showed blood congestion, various degrees of lamellar hyperplasia, including complete fusion of lamellae, as well as hypertrophy and hyperplasia of chloride and mucous cells in white seabass (*Lates calcarifer*), catla (*Labeo catla*) and Nile tilapia [97, 98, 99]. In the present study, at much lower exposure concentrations, and similarly to copper, hyperemia of the secondary lamellae was significantly higher in the group exposed to the lower concentration. Ultrastructural studies showed that cadmium is responsible for enlargement of microridges in epithelial cells, but it also has an influence on chloride cells density and their apical area [100].

Contrary to copper and cadmium, studies of zinc toxicity to fish gills and their subsequent morphological changes remained scarce. The exposure concentrations for zinc are relatively low in comparison to the high background levels in the gill tissue. This, in combination with the short exposure time not allowing zinc to accumulate up to statistically different levels among treatment groups, explains the low degree of histological effects. Nevertheless, in both zinc groups, oedema of the primary epithelium was significantly higher compared to the control group. This was also found in a study where *Astyanax* aff. *bimaculatus* was exposed to higher concentrations of zinc [101], and was also noted in cichlid fish (*Etroplus suratensis*), upon exposure to zinc [87]. This is most likely a consequence of disrupted calcium homeostasis [102] as a direct consequence of competition at the uptake site, the epithelial calcium channel, or because of influence of zinc on the cellular Ca^2+^ signaling [103, 104, 105], although we did not measure significant drops in gill calcium levels here.

Two alterations that contributed the most to the highest levels of the total gill index score in our study are oedema and structural and architectural alterations. Both changes are not of high histopathological significance, and could easily be reverted when the stressor is removed from the aquatic environment [106]. Concerning the specificity of histopathological changes, oedema of the primary epithelium was the only alteration absent in the control and both groups exposed to copper, but showed significantly higher scores in fish exposed to Cd_50_, Zn_10_ and Zn_50_. Lamellar oedema is originating as a consequence of ultrafiltration subjected to increased arterial blood pressure, and [107] compared this mechanism to glomerular filtration in the kidney. The described process is easily reversible and if stressor is removed from the water, this will happen fairly rapid [108]. Cadmium and zinc share common mechanisms of toxicity [109] and this is probably the explanation for the absence of oedema in the copper exposed fish. Fish exposed to higher concentrations of copper and cadmium were exhibiting increased scores in regressive changes. Regressive changes are defined as “malformation or dysfunction of cellular structures as a result of cell damage” [41] and this pattern is generally linked to a decreasing number of cells in the tissue, contrary to progressive changes.

## Conclusion

In conclusion, our acute exposure showed that bioaccumulation of copper and cadmium was dose dependent, but that this was not the case for zinc. Furthermore, only cadmium bioaccumulation showed a clear correlation with mortality. This pattern was confirmed in the sublethal exposures at equitoxic exposure concentrations. We hypothesized that metal specific alterations would occur, but these appeared to be rather limited. Copper did interfere with tissue sodium levels as hypothesized, but cadmium and zinc did not interfere with tissue calcium levels. Histopathological changes at both 10 and 50% of the LC50 value were mild for all metals and mostly general in nature (e.g. hyperemia and hypertrophy) with exception of oedema of the primary epithelium which could be linked to zinc at both exposure levels, and to a lesser extent to cadmium. It would have been interesting to examine a wider variety of physiological parameters, to assess which metal specific processes are involved at equally toxic exposures. Now that baseline toxicity data under equal exposure conditions are available, further studies could be directed at more chronic sublethal equitoxic exposures, and include a broader scale of physiological parameters not only linked to ionoregulation, but also to olfactory responses, immunity, oxidative stress and protective protein such as metallothionein, as these processes can play an important role as well. Subsequently, to increase environmental relevance, the effects of metal mixtures should be assessed, as negative additive or synergistic effects could occur.

## Supporting information

S1 Data (XLSX)
